# Structure and Wear Performance of a Titanium Alloy by Using Low-Temperature Plasma Oxy-Nitriding

**DOI:** 10.3390/ma16103609

**Published:** 2023-05-09

**Authors:** Haidong Li, Haifeng Wang, Shijie Wang, Yange Yang, Yunsong Niu, Shenglong Zhu, Fuhui Wang

**Affiliations:** 1 AVIC Xi’an Flight Automatic Control Research Institute, Xi’an 710065, China; 2 Shi-Changxu Innovation Center for Advanced Materials, Institute of Metal Research, Chinese Academy of Sciences, Shenyang 110016, China; 3 Shenyang National Key Laboratory for Materials Science, Northeastern University, Shenyang 110819, China

**Keywords:** plasma oxy-nitriding, titanium alloy, low-temperature, thick coating

## Abstract

To solve the problems of high nitriding temperature and long nitriding time with conventional plasma nitriding technologies, a kind of low-temperature plasma oxy-nitriding technology containing two-stage processes with different ratios of N to O was developed on a TC4 alloy in this paper. A thicker permeation coating can be obtained with this new technology compared to conventional plasma nitriding technology. The reason for this is that the oxygen introduction in the first two-hour oxy-nitriding step can break the continuous TiN layer, which facilitates the quick and deep diffusion of the solution-strengthening elements of O and N into the titanium alloy. Moreover, an inter-connected porous structure was formed under a compact compound layer, which acts as a buffer layer to absorb the external wear force. Therefore, the resultant coating showed the lowest COF values during the initial wear state, and almost no debris and cracks were detected after the wear test. For the treated samples with low hardness and no porous structure, fatigue cracks can easily form on the surface, and bulk peeling-offcan occur during the wear course.

## 1. Introduction

Metals, including many kinds of composites, are greatly popular in modern industry [1,2,3,4,5,6,7,8]. Among them, titanium and its alloys are widely used in aircraft, aerospace, and marine equipment due to their light weight, high specific strength, and excellent corrosion resistance [9,10,11,12]. However, they are susceptible to abrasion because of low wear resistance, which is their principal drawback [13,14]. Therefore, surface-hardening titanium alloys with hardened layers tens to hundreds of microns thick can benefit significantly from the application of a kind of substrate-strengthening thermochemical diffusion treatment: plasma nitriding [15,16,17].

Plasma nitriding favors the formation of hard and wear-resistant ceramic compound layers of titanium nitrides on titanium and its alloys, as well as a solid-solution sublayer of N-interstitial α-Ti crystals [18]. High-temperature nitriding with a long time of treatment can significantly improve the hardness and wear performance of titanium alloys [18,19,20] but can cause mechanical degradation, unpredictable deformation, and even β phase transformation leading to failure of the industrial components [21]. As a probable method to overcome these drawbacks, low-temperature plasma nitriding treatment below 700 °C has attracted the attention of many researchers [22,23,24,25,26]. However, plasma nitriding at low temperatures cannot produce deep nitrogen diffusion into substrates due to tardy nitriding kinetics for the diffusion-controlled feature [27,28]. Moreover, Tarnowski [16] and Fu [25] reported that low-temperature plasma nitriding can easily yield transverse cracks into substrates at low external loads. Sun [29] found that crack initiation can fast lead to rapid fracturing, resulting from the crack propagation behavior spreading into the substrate interior. Therefore, it is important to promote the diffusion rate of nitrogen and to protect the mechanical properties of parts but not simply by reducing the temperature and prolonging the nitriding duration time. Essentially, the main reason for the low thickness of the diffusion layer and the low hardness of workpieces at low temperatures is the formation of a continuous Ti_2_N or TiN layer on the titanium surface, which inhibits nitrogen diffusion as an element barrier [30].

Another promising way to further improve the wear performance of titanium alloys is the formation of TiN_x_O_y_ [31]. TiN_x_O_y_ possesses higher microhardness and wear resistance, which exceeds the properties of single TiN_x_ or TiO_x_ [32,33]. Thermal oxidation is a kind of strengthening surface-modified process in air nevertheless leading to easy severe oxide stratification. Oxygen-boosted diffusion can produce quick and deep case hardening into titanium through vacuum annealing due to the TiO_2_ inner dissolution mechanism and avoiding nitrogen accumulation at the oxide/titanium interface, which can inhibit the boost diffusion of the subsequent element [34]. In other words, one can conclude that oxygen-boosted deep diffusion can occur in low-vacuum and low-temperature conditions to avert mechanical degradation at high temperatures and during long-time treatment.

Therefore, this work is aimed at developing a novel and efficient plasma compound oxy-nitriding technology at a low temperature of 650 °C to obtain deep penetration of solid solution strengthening elements of N and O into a TC4 alloy. Moreover, through the plasma oxy-nitriding course, a kind of special microstructure in the compound layer was developed to avoid crack initiation during the wear process. In this paper, the mechanism of how to penetrate deep into the substrate and the corresponding energy-absorbing function of its microstructure in the coating by this novel oxy-nitriding technology are further discussed.

## 2. Materials and Methods

The material used in this research is a Ti6Al4V substrate. The specimens were cut to a size of 20 × 20 × 6 mm^3^. In order to achieve a fine finish, all the specimens were ground using SiC abrasive paper up to 2000 mesh grit, ultrasonically degreased in acetone for 10 min, and dried before plasma oxy-nitriding treatment.

The specimens were placed into a chamber evacuated below 5 Pa and then treated with plasma cleaning at an argon partial pressure of 15 Pa for 20 min. After that, five different surface treatments were conducted on TC4 alloy specimens at 650 °C, and the detailed process parameters of different processes are listed in Table 1. For the PN3h sample, it is a conventional plasma nitride treatment for 3 h and the other four are plasma oxy-nitride samples with two-step treatments. Specifically, PON3h-30Pa, PON3h-35Pa, and PON3h-40Pa are oxy-nitride samples for 3 h with different nitrogen partial pressures in the second step, while PON4h-40Pa is the only one with a total plasma treatment time for 4 h with the first oxy-nitriding step for 2 h.

The surface and cross-section morphologies of the oxy-nitrided samples were observed by a scanning electron microscope (SEM, TESCAN MIRA3, Czech Republic) with an accelerating voltage of 20 kV. Transmission electron microscopy (TEM, FEI Talos F200X, USA) was used for microstructure and composition analysis at an accelerating voltage of 200 kV. The phase constituents were identified by X-ray diffraction (XRD, PANalytical X’Pert Pro, The Netherlands) with Cu-*Ka* (λ = 1.54 Å) radiation, at a scanning range of 10~90° and an accelerating voltage of 40 kV, a generator of 40 mA, and a scanning step of 0.1°. The microhardness of the oxy-nitrided coatings was measured using an microhardness tester (HV-1000 Vickers, China) at a load of 25 g and a duration time of 15 s. The wear test was explored on a reciprocating friction machine (Rtec MFT-5000, USA) at room temperature to investigate the dry-sliding wear performance of the coatings. A GCr15 ball, with a diameter of 4 mm, was loaded as the stationary pin. The load, frequency, and total wear time were 10 N, 2 Hz, and 10 min, respectively. The nano-hardness and elastic modulus of the samples were measured by a G200 nanoindentation system equipped with a Berkovich tip.

## 3. Results and Discussion

**Figure 1:**
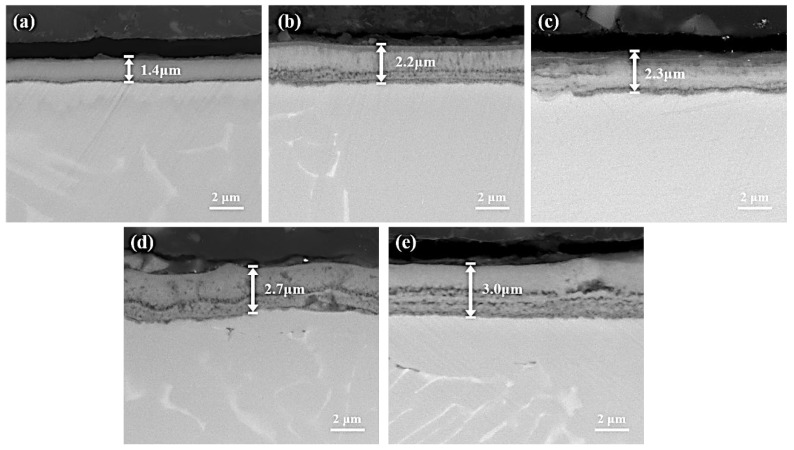
Cross-sectional microstructure of the samples obtained by five different processes: (**a**) PN3h, (**b**) PON3h-30Pa, (**c**) PON3h-40Pa, (**d**) PON3h-40Pa, and (**e**) PON4h-40Pa.

Cross-sectional micrographs of the titanium alloy with five different processes are shown in Figure 1. In Figure 1a, the plasma nitride sample shows the classical well-pronounced compound layer on the surface. Different from it, the oxy-nitrided samples (Figure 1b–e) show an obvious two-layer structure with a dense outer layer and a loose inner layer. The thickness of the compound layer for the oxy-nitrided samples was larger than that of the plasma nitride sample. Moreover, the thickness was further increased as the nitrogen partial pressure and plasma oxy-nitriding time increased.

**Figure 2:**
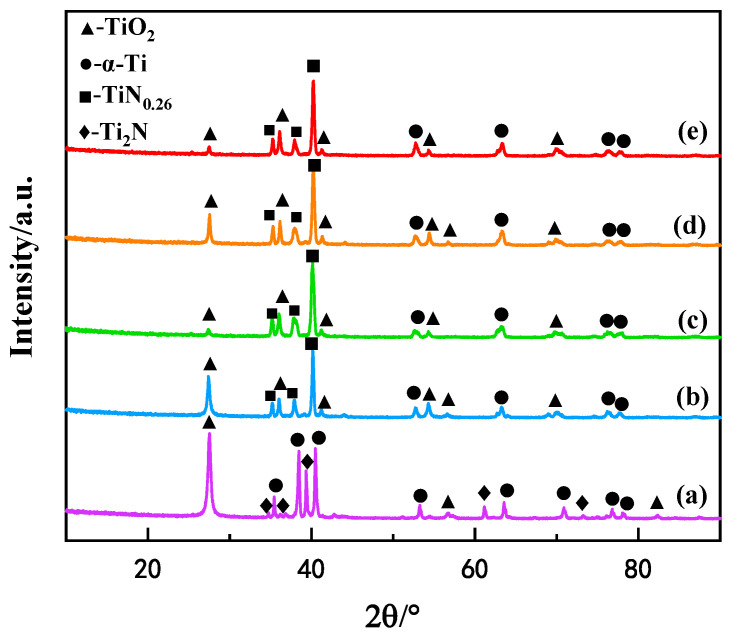
XRD patterns of samples obtained by five different processes: (**a**) PN3h, (**b**) PON3h-30Pa, (**c**) PON3h-35Pa, (**d**) PON3h-40Pa, and (**e**) PON4h-40Pa.

Figure 2 presents the diffraction patterns of the TC4 alloy samples obtained by five different processes. In addition, the average grain size for each sample was calculated. For the plasma nitrided sample with an average grain size of 27 nm, the phase compositions consisted of α-Ti(N) (PDF#44-1294), Ti_2_N (PDF#17-0386), and TiO_2_ (PDF#21-1276). The residual oxygen in the chamber was involved in the plasma nitriding process, and the oxygen ion cannot penetrate the as-formed Ti_2_N compound layer, so they react with TiN to form TiO_2_. That is the reason for the high intensity of the TiO_2_ peak in the nitrided samples at 27.446°. Differently to the nitrided samples, the phase compositions of the oxy-nitrided samples are composed of α-Ti(N), TiN_0.26_ (PDF#44-1095), and rutile TiO_2_. The average grain sizes are 29 nm, 18 nm, 22 nm, and 21 nm for the PON3h-30Pa, PON3h-35Pa, PON3h-40Pa, and PON4h-40Pa samples, respectively. It can be seen that weaker but more numerous peaks of the rutile TiO_2_ phase were detected. That is because during the first oxy-nitriding course, the partial pressure of oxygen reached 3 Pa and its proportion in the reaction gas is about 25%, which is much greater than that of the nitrided sample. More oxygen ions can be preferentially adsorbed on the surface of the titanium alloy and continuously diffuse deep into the titanium alloy. In other words, the oxygen atoms were not agglomerated on the surface like the nitrided samples but were submerged inside the alloy, so that weak and multiple TiO_2_ peaks could be detected for the oxy-nitrided samples. Moreover, the Ti_2_N phase disappeared but TiN_0.26_ emerged in the oxy-nitriding samples. This is another key disparity between the nitriding and oxy-nitriding processes.

**Figure 3:**
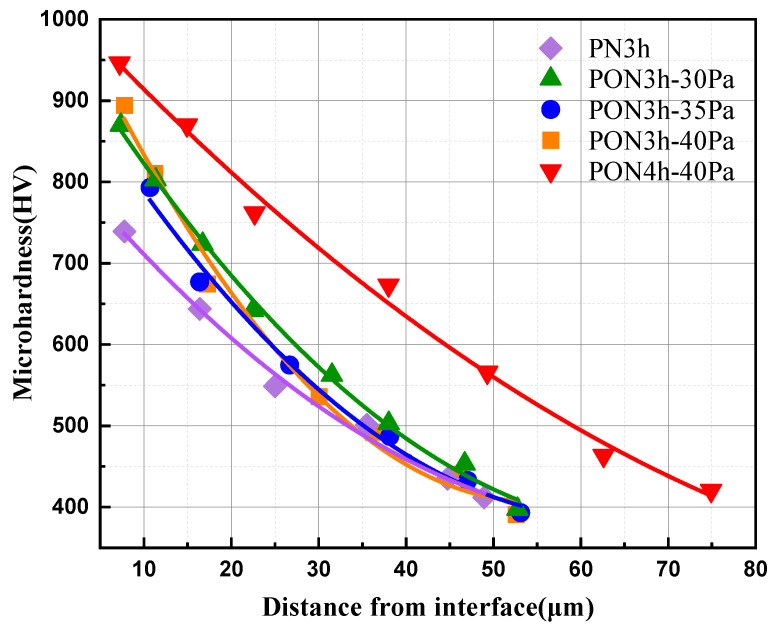
Hardness vs. depth for different plasma treatment processes.

The microhardness of the titanium alloy with different treatments along the cross-section is shown in Figure 3. The plasma nitrided treatment produced a total hardened layer of about 52 μm in depth for the PN3h sample. The thickness of the hardened layer for oxy-nitrided samples (PON3h-30Pa, PON3h-35Pa, and PON3h-40Pa) was the same as the nitride sample and was not unchanged with increasing nitrogen partial pressure. However, the increased hardness at the same depth indicates that more N and O are incorporated into the titanium matrix compared to the PN3h sample. Furthermore, both the thickness of the hardened layer and the hardness at the same depth of the titanium alloy were obviously increased by comparing sample PON3h-40Pa with PON4h-40Pa. The thickness of the hardened layer for the PON4h-40Pa sample reached 75 μm. In summary, the developed plasma oxy-nitriding technology could improve the hardness of the diffusion layer compared with the conventional plasma nitriding technology, and the oxy-nitriding time in the first step was the key parameter to obtain a deep hardened layer for the titanium alloy.

**Figure 4:**
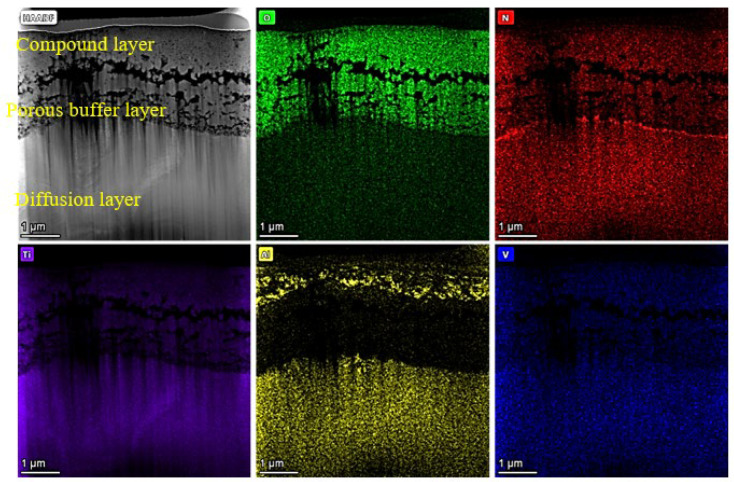
Cross-section and element distribution of the PON4h-40Pa sample characterized by TEM.

The cross-section and element distribution of the PON4h-40Pa sample were further characterized by TEM and are shown in Figure 4. Firstly, a porous layer was observed between the compound layer and the diffusion layer, which is different from the conventional plasma nitriding treatment. Secondly, beneath the porous structure, there is a thin N-rich layer with many N-broken hemi-continuous passageways. Sun [30] reported that TiN can act as the diffusion barrier layer to prevent elemental diffusion at temperatures up to 700 °C. For the PON4h-40Pa sample treated at 650 °C, TiN_0.26_ was produced as shown in Figure 2, indicating that the continuous TiN layer was broken due to the introduction of O in this work. No continuous and integrated TiN layer can be formed to block the fast diffusion of the strengthening elements. These N breakpoints via oxygen introduction provided a pathway for the subsequent diffusion of N and O accessing the TC4 substrate and resulted in the thickest hardened layer.

Here, an O-doped atmosphere was used to ruin the TiN barrier effect, so that deep penetration of O and N could be obtained by prolonging the first oxy-nitriding course. The number of facilitating passageways for the PON3h-30Pa, PON3h-35Pa, and PON3h-40Pa samples is reduced resulting from their short action time in the first oxy-nitriding course. Accordingly, the inapparent thickness increase in the hardened layer can be obtained after double action time in the first oxy-nitriding course for the PON4h-40Pa sample. Moreover, an interconnected porous layer can be observed below the outermost dense layer. However, no TiO_x_N_y_ peaks can be detected from the XRD results. Hence, it is a kind of N-doped TiO_2_ material that is formed on the TC4 surface [35,36], not the TiO_x_N_y_ ceramics.

**Figure 5:**
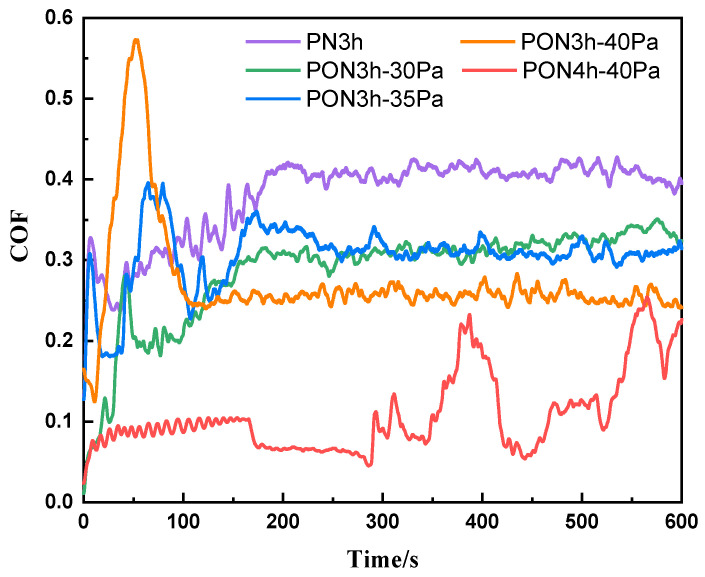
The COF versus the sliding time for the coating samples obtained by five different processes.

**Figure 6:**
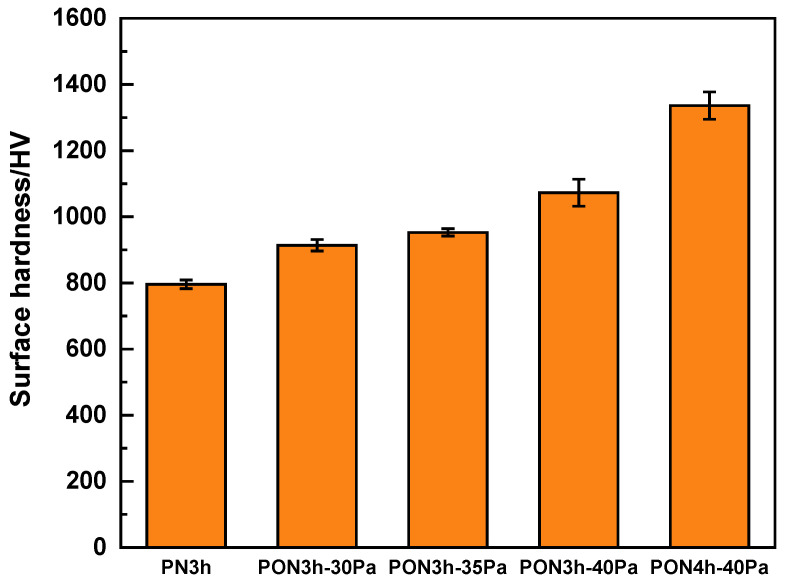
The surface hardness of the samples obtained by five different processes.

**Figure 7:**
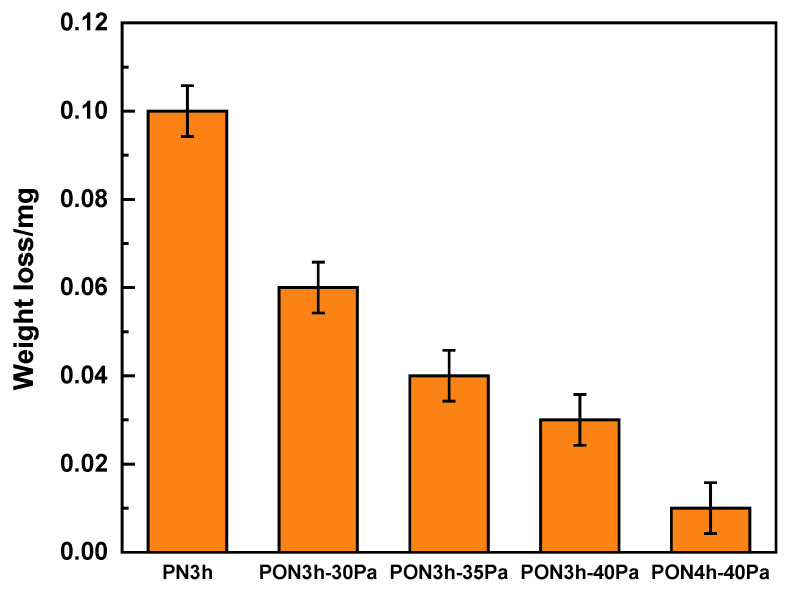
The weight loss of the samples obtained by five different processes after the wear test.

The coefficient of friction (COF) vs. the time plot of the samples with five different processes is disclosed in Figure 5. Initially, the COF values for all five samples experience violent turbulence before reaching a steady-state condition. A fast increase and a sharp decline of the PN3h sample within 10 s mean that the GCr15 ball has conquered the compound layer and already passed into the diffusion layer. Then, the comparatively gradual increase in COF from 10 s to about 200 s is associated with mutual plowing between GCr15 and the diffusion layer, due to surface roughening and trapped wear particles [37]. Finally, the value becomes stable at 0.4 as the increase in wear track roughness has reached the maximum value. This is a conventional wear procedure. As for the oxy-nitrided samples with one-hour oxy-nitriding in the first course, a noticeable fluctuation (slow increase and subsequent decrease) in the COF occurred, indicating that the initial state occurs based on the rough and inhomogeneous surface [38], in coincidence with the TEM observation in Figure 4a, which is composed of a compact compound layer and a porous interlayer for the PON3h-30Pa, PON3h-35Pa, and PON3h-40Pa samples. In addition, the COF in the steady state is decreased with the increase in nitrogen partial pressure. However, the PON4h-40Pa sample shows a significantly sustained lower COF than the other oxy-nitrided samples. In addition, the inter-connected porous layer acted as a buffer layer which may absorb the friction force and thus reduce the plow-in effect. Both the surface hardness as shown in Figure 6 and the special microstructure of the plasma layers led to a drastic decrease in the COF value. Moreover, the mass loss in the average after-wear test of the samples is shown in Figure 7, further proving the continuous plow-in decreasing effect for the five different processes. In addition, the hard-oxide-mixed wear debris, i.e., TiO_2_ and Al_2_O_3_ (see the elementary mapping in Figure 4), generated in the oxy-nitriding course, playing a part in third-body lubricity, can decrease both the wear loss and the coefficient of friction. Zhu [39] reported that the presence of oxide would reduce the surface roughness, thereby improving the smoothness of the worn surface to reduce the COF. Moreover, Huang [40] reported that TiO_2_ can also play a role in load-bearing capacity, resulting in friction-reduction behavior and a reduction in the self-lubricating effect on the size of debris. The long-time low COF values can be ascribed to the synergistic effect of high hardness, the buffering porous structure with enough space, and the self-lubricating function of TiO_2_.

**Figure 8:**
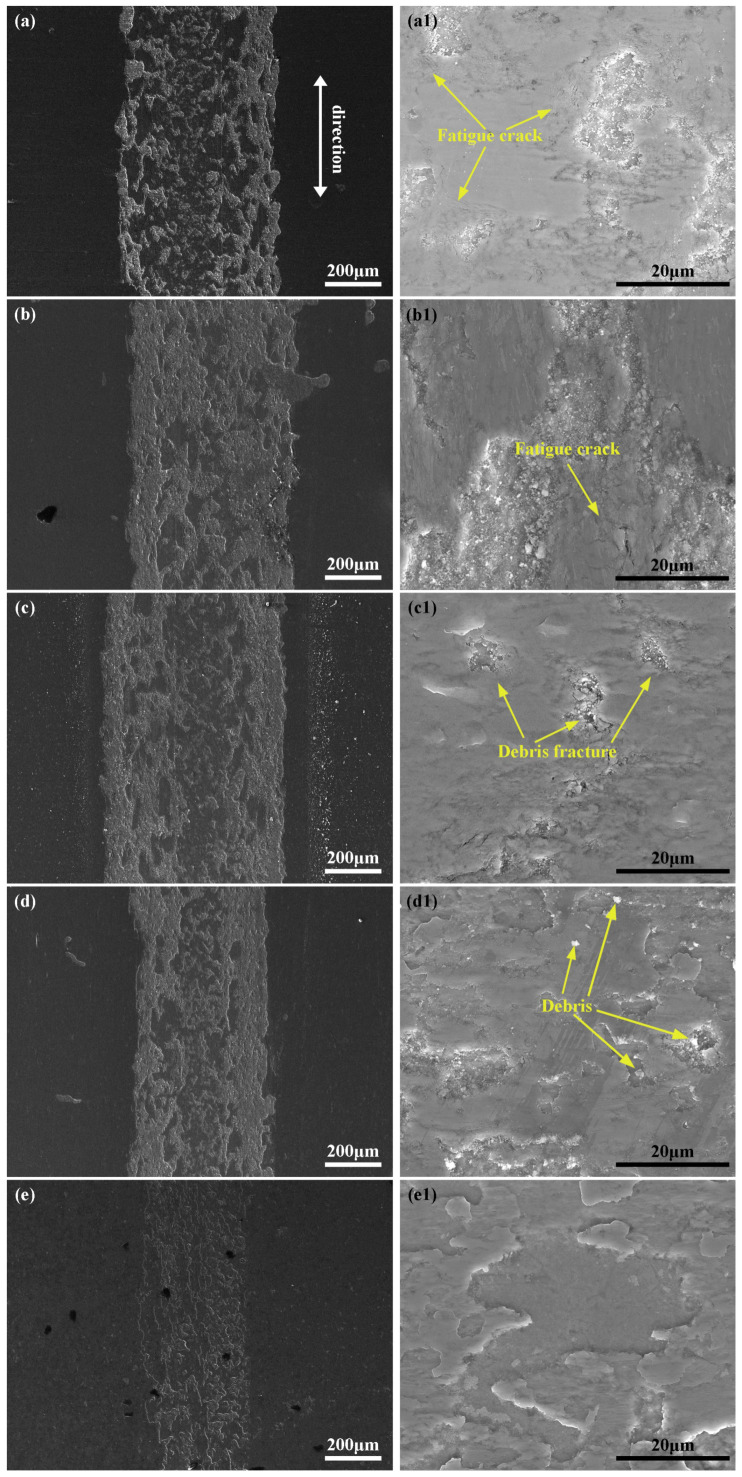
Micro-morphologies of TC4 samples obtained by five different processes: (**a**,**a1**) PN3h, (**b**,**b1**) PON3h-30Pa, (**c**,**c1**) PON3h-40Pa, (**d**,**d1**) PON3h-40Pa, and (**e**,**e1**) PON4h-40Pa.

Figure 8 shows the wear tracks of the five worn sample surfaces and it can be seen that adhesive wear is the predominant form of damage for all samples. In order to detect the cracks, the worn morphologies at high magnification were employed. Large amounts of fatigue cracks can be seen on the surface of the PN3h sample, which further proved the easy crack initiation for the nitrided samples. Treated by our oxy-nitrided technology, cracks cannot be easily detected from the worn morphologies except for the PON3h-30Pa sample, due to the lower nitrogen partial pressure and hardness of the resultant plasma layer, which cannot endure the long-time wear procedure and yields to crack initiations. With the increasing nitrogen partial pressure, the surface hardness significantly improved, which can bear the external load for a comparatively longer time. Some debris fracture behavior can also be observed for the PON3h-35Pa and PON3h-40Pa samples as seen in Figure 8c1,d1. Correspondingly, the crushed particles were the result of the detaching behavior from the previous cohesive wear debris and were milled into a free state with no binding force with the downward sample surfaces. Hence, they can be easily swept away by the following back-and-forth movement of the friction pair and separated with both friction pairs thoroughly. However, prolonging the time in the first oxy-nitrided course, the complete elimination of crack and debris happened for the PON4h-40Pa sample, due to not only the ultra-high hardness but also the buffering effect resulting from the special porous structure of the layer, which can bring enough elastic deformation against the wear force. Therefore, the wear mechanism for the PON4h-40Pa sample is contact fatigue with the buffering effect of enough inter-connected porous structures.

**Figure 9:**
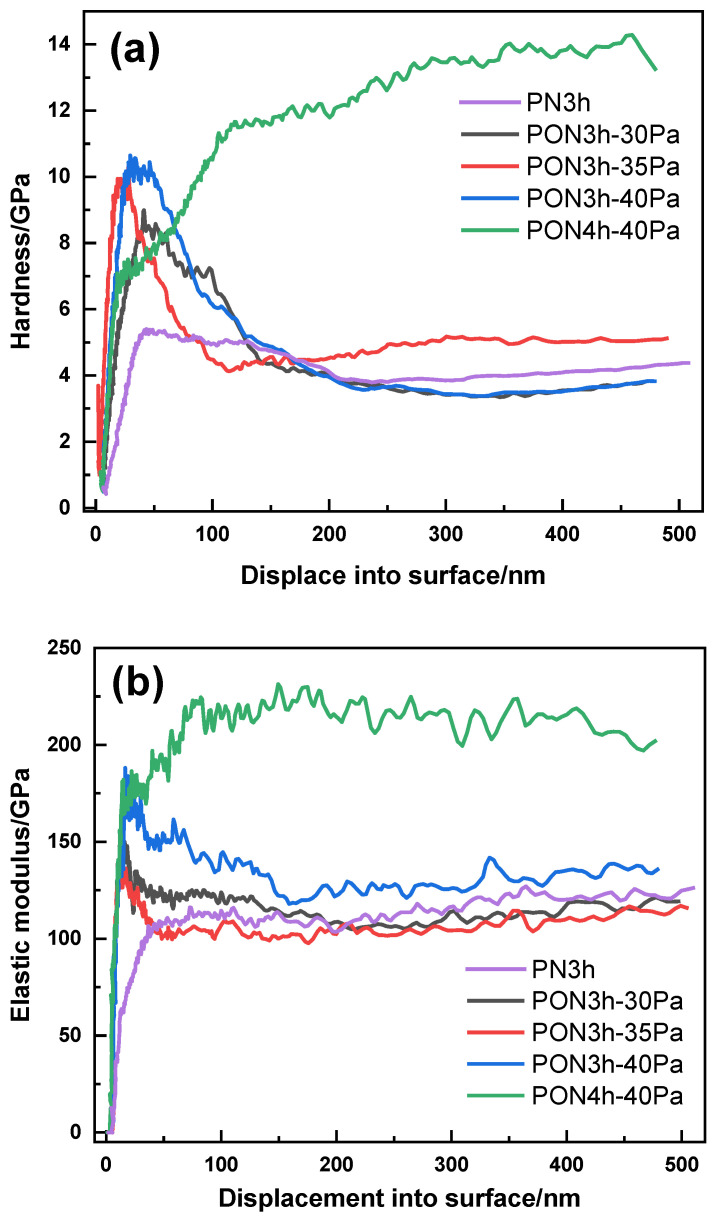
Hardness and elastic modulus with the indentation depth for the samples obtained by five different processes: (**a**) hardness and (**b**) elastic modulus.

In order to further illustrate the buffering effect of the porous structure, Figure 9 displays the hardness and elastic modulus of the layers measured by nanoindentation for the samples with five different processes. Figure 9 clearly shows that an apparent hardness and elastic moduli exhibit the initial fast increase with the increasing indent load, which is attributed to the surface roughness being comparable to the indentation depth at low loads that can underestimate E and H values at shallow indentation depths [41]. Such an effect can be eliminated with the depth penetrating, leading to a leveling-off at higher loads, except for the PON4h-40Pa sample. In principle, densification in materials, which is generated by crushing the porous structure under the indenter rather than the conventional plastic deformation could considerably increase the local elastic modulus and hardness. The proper porosity density played a significant role in absorbing the external force, which can reduce the strain sensitivity of quick plastic deformation for coatings [42]. Therefore, the nitrided sample (PN3h) shows the quickest measuring stability, indicating the high sensitivity of the external force. This can further testify to the long-time low COF behavior of the oxy-nitrided sample (PON4h-40Pa).

## 4. Conclusions

A novel kind of plasma oxy-nitriding technology at 650 °C was developed in this paper and a thicker permeation coating was obtained on a TC4 alloy compared to conventional plasma nitriding technology. The best process contained two steps oxy-nitriding processes with different ratios of N to O. The introduction of oxygen in the first oxy-nitriding process can prevent the formation of a continuous TiN barrier layer. The breakpoint of the discontinuous TiN layer provided a pathway for the subsequent quick permeation of O and N into the titanium alloy. For the oxy-nitrided sample with 2 h treatment in the first step, an inter-connected porous structure under a compact compound layer was formed, which acts as a buffer layer to absorb the external force. Therefore, the wear performance of the oxy-nitrided sample with a special microstructure showed the lowest COF value during the initial wear state, and almost no debris and cracks were detected after the wear test. On the contrary, for the samples with low hardness and no porous structure, fatigue cracks can be easily formed on the surface and bulk peeling-off can occur during the wear course. The inter-connected porous structure and high hardness play a key role in the anti-wear course. Moreover, the oxide deeply penetrated the diffusion layer. TiO_2_, as a kind of high-temperature self-lubricated phase, may exhibit excellent high-temperature wear behavior and will be further investigated in the future.

## Figures and Tables

**Table 1:** Detailed parameters of the different processes.

Sample	First Oxy-Nitriding Course			Second Oxy-Nitriding Course		
	O_2_:N_2_	Pressure (Pa)	Time (h)	O_2_:N_2_	Pressure (Pa)	Time (h)
PN3h	\	\	\	100% N_2_	40	3
PON3h-30Pa	1:4	15	1	1:9	30	2
PON3h-35Pa	1:4	15	1	1:11	35	2
PON3h-40Pa	1:4	15	1	1:13	40	2
PON4h-40Pa	1:4	15	2	1:13	40	2

## Data Availability

Datas are available on reasonable request from the corresponding author.

## References

[1] Tayyebi M., Alizadeh M. (2023). Thermal and wear properties of Al/Cu functionally graded metal matrix composite produced by severe plastic deformation method. J. Manuf. Process..

[2] Ye Q., Li X., Tayyebi M., Assari A.H., Polkowska A., Lech S., Polkowski W., Tayebi M. (2023). Effect of heat treatment parameters on microstructure evolution, tensile strength, wear resistance, and fracture behavior of Ni–Ti multilayered composites produced by cross-accumulative roll bonding. Arch. Civ. Mech. Eng..

[3] Mohazzab B.F., Jaleh B., Fattah-alhosseini A., Mahmoudi F., Momeni A. (2020). Laser surface treatment of pure titanium: Micro-structural analysis, wear properties, and corrosion behavior of titanium carbide coatings in Hank’s physiological solution. Surf. Interfaces.

[4] Xu M., Yu X., Zhang S., Yan S., Tarbokov V., Remnev G., Le X. (2023). Microstructure Formation and Mechanical Properties of Metastable Titanium-Based Gradient Coating Fabricated via Intense Pulse Ion Beam Melt Mixing. Materials.

[5] Gloc M., Przybysz-Gloc S., Wachowski M., Kosturek R., Lewczuk R., Szachogłuchowicz I., Paziewska P., Maranda A., Ciupiński Ł. (2023). Research on Explosive Hardening of Titanium Grade 2. Materials.

[6] Pintilei G.L., Crismaru V.I., Abrudeanu M., Munteanu C., Luca D., Istrate B. (2015). The influence of ZrO_2_/20%Y_2_O_3_ and Al_2_O_3_ deposited coatings to the behavior of an aluminum alloy subjected to mechanical shock. Appl. Surf. Sci..

[7] Baltatu M.S., Sandu A.V., Nabialek M., Vizureanu P., Ciobanu G. (2021). Biomimetic Deposition of Hydroxyapatite Layer on Titanium Alloys. Micromachines.

[8] Wang Y., Tayyebi M., Assari A. (2022). Fracture toughness, wear, and microstructure properties of aluminum/titanium/steel multi-laminated composites produced by cross-accumulative roll-bonding process. Arch. Civ. Mech. Eng..

[9] Yumusak G., Leyland A., Matthews A. (2020). The effect of pre-deposited titanium-based PVD metallic thin films on the nitrogen diffusion efficiency and wear behaviour of nitrided Ti alloys. Surf. Coat. Technol..

[10] Genc O., Unal R. (2022). Development of gamma titanium aluminide (γ-TiAl) alloys: A review. J. Alloys Compd..

[11] Guo A.X., Cheng L., Zhan S., Zhang S., Xiong W., Wang Z., Wang G., Cao S.C. (2022). Biomedical applications of the powder-based 3D printed titanium alloys: A review. J. Mater. Sci. Technol..

[12] Chirico C., Romero A.V., Gordo E., Tsipas S. (2022). Improvement of wear resistance of low-cost powder metallurgy β-titanium alloys for biomedical applications. Surf. Coat. Technol..

[13] Yumusak G., Leyland A., Matthews A. (2022). A microabrasion wear study of nitrided α-Ti and β-TiNb PVD metallic thin films, pre-deposited onto titanium alloy substrates. Surf. Coat. Technol..

[14] Zhao S., Meng F., Fan B., Dong Y., Wang J., Qi X. (2023). Evaluation of wear mechanism between TC4 titanium alloys and self-lubricating fabrics. Wear.

[15] Wen K., Zhang C., Gao Y. (2022). Influence of gas pressure on the low-temperature plasma nitriding of surface-nanocrystallined TC4 titanium alloy. Surf. Coat. Technol..

[16] Tarnowski M., Borowski T., Skrzypek S., Kulikowski K., Wierzchoń T. (2021). Shaping the structure and properties of titanium and Ti6Al7Nb titanium alloy in low-temperature plasma nitriding processes. J. Alloys Compd..

[17] Mohan L., Anandan C. (2013). Effect of gas composition on corrosion behavior and growth of apatite on plasma nitrided titanium alloy Beta-21S. Appl. Surf. Sci..

[18] Bacci T., Borgioli F., Tesi B. (1998). Surface modification of Ti–6Al–4V alloy by means of combined plasma nitriding and oxidising treatments. Surf. Eng..

[19] Kikuchi S., Ota S., Akebono H., Omiya M., Komotori J., Sugeta A., Nakai Y. (2016). Formation of nitrided layer using atmospheric-controlled IH-FPP and its effect on the fatigue properties of Ti-6Al-4V alloy under four-point bending. Procedia Struct. Integr..

[20] Takesue S., Kikuchi S., Akebono H., Morita T., Komotori J. (2020). Characterization of surface layer formed by gas blow induction heating nitriding at different temperatures and its effect on the fatigue properties of titanium alloy. Results Mater..

[21] Shen H., Wang L. (2020). Formation, tribological and corrosion properties of thicker Ti-N layer produced by plasma nitriding of titanium in a N2-NH3 mixture gas. Surf. Coat. Technol..

[22] She D., Yue W., Fu Z., Wang C., Yang X., Liu J. (2015). Effects of nitriding temperature on microstructures and vacuum tribological properties of plasma-nitrided titanium. Surf. Coat. Technol..

[23] Hacısalihoğlu İ., Kaya G., Ergüder T.O., Mandev E., Manay E., Yıldız F. (2021). Tribological and thermal properties of plasma nitrided Ti45Nb alloy. Surf. Interfaces.

[24] Borisyuk Y., Oreshnikova N., Berdnikova M., Tumarkin A., Khodachenko G., Pisarev A. (2015). Plasma Nitriding of Titanium Alloy Ti5Al4V2Mo. Phys. Procedia.

[25] Fu Y.-D., Zhu X.-S., Li Z.-F., Leng K. (2016). Properties and microstructure of Ti6Al4V by deformation accelerated low temperature plasma nitriding. Trans. Nonferr. Met. Soc. China.

[26] Farokhzadeh K., Edrisy A., Pigott G., Lidster P. (2013). Scratch resistance analysis of plasma-nitrided Ti–6Al–4V alloy. Wear.

[27] Zhecheva A., Sha W., Malinov S., Long A. (2005). Enhancing the microstructure and properties of titanium alloys through nitriding and other surface engineering methods. Surf. Coat. Technol..

[28] Chen K., Jaung G.D.c. (1997). diode ion nitriding behavior of titanium and Ti-6Al-4V. Thin Solid Films.

[29] Sun B., Wang L., Sun Y., Gao J., Cao H., Ren J., Cui J., Yuan X., Li A., Wang C. (2021). Enhanced thermal stability of Mo film with low infrared emissivity by a TiN barrier layer. Appl. Surf. Sci..

[30] Wang C., Chen W., Chen M., Chen D., Yang K., Wang F. (2020). Effect of TiN diffusion barrier on elements interdiffusion behavior of Ni/GH3535 system in LiF-NaF-KF molten salt at 700 °C. J. Mater. Sci. Technol..

[31] Rizzo A., Signore M.A., Mirenghi L., Di Luccio T. (2009). Synthesis and characterization of titanium and zirconium oxynitride coatings. Thin Solid Films.

[32] Pohrelyuk I., Morgiel J., Tkachuk O., Szymkiewicz K. (2019). Effect of temperature on gas oxynitriding of Ti-6Al-4V alloy. Surf. Coat. Technol..

[33] Dong H., Li X. (2000). Oxygen boost diffusion for the deep-case hardening of titanium alloys. Mater. Sci. Eng. A.

[34] Jadhav P.S., Jadhav T., Bhosale M., Jadhav C., Pawar V. (2021). Structural and optical properties of N-doped TiO_2_ nanomaterials. Mater. Today Proc..

[35] Wang Y., Tan Q., Huang B. (2021). Synthesis and properties of novel N/Ta-co-doped TiO_2_ coating on titanium in simulated PEMFC environment. J. Alloys Compd..

[36] Sayegh S., Abid M., Tanos F., Cretin M., Lesage G., Zaviska F., Petit E., Navarra B., Iatsunskyi I., Coy E. (2022). N-doped TiO_2_ nanotubes synthesized by atomic layer deposition for acetaminophen degradation. Colloids Surf. A.

[37] Gao W., Li Z. (2008). 6—Tribological degradation at elevated temperature. Developments in High Temperature Corrosion and Protection of Materials.

[38] Allahyarzadeh M., Aliofkhazraei M., Rouhaghdam A.S., Alimadadi H., Torabinejad V. (2020). Mechanical properties and load bearing capability of nanocrystalline nickel-tungsten multilayered coatings. Surf. Coat. Technol..

[39] Zhu Y., Liu X.B., Liu Y.F., Wang G., Wang Y., Meng Y., Liang J. (2021). Development and characterization of Co-Cu/Ti_3_SiC_2_ self-lubricating wear resistant composite coatings on Ti6Al4V alloy by laser cladding. Surf. Coat. Technol..

[40] Huang Z.P., Zhao W.J. (2020). Coupling hybrid of HBN nanosheets and TiO_2_ to enhance the mechanical and tribological properties of composite coatings. Prog. Org. Coat..

[41] Chen Z., Wang X., Bhakhri V., Giuliani F., Atkinson A. (2013). Nanoindentation of porous bulk and thin films of La_0.6_Sr_0.4_Co_0.2_Fe_0.8_O_3−δ_. Acta Mater..

[42] Chen W., Wang H. (2021). Reduced strain rate sensitivity by structural rejuvenation in metallic glass under nanoindentation. Mater. Lett..

